# Identification and Characterization of a New Erythromycin Biosynthetic Gene Cluster in *Actinopolyspora erythraea* YIM90600, a Novel Erythronolide-Producing Halophilic Actinomycete Isolated from Salt Field

**DOI:** 10.1371/journal.pone.0108129

**Published:** 2014-09-24

**Authors:** Dandan Chen, Junyin Feng, Lei Huang, Qinglin Zhang, Jiequn Wu, Xiangcheng Zhu, Yanwen Duan, Zhinan Xu

**Affiliations:** 1 Department of Chemical and Biological Engineering, Zhejiang University, Hangzhou, China; 2 Huzhou Center of Bio-synthetic Innovation, Shanghai Institute of Organic Chemistry, Chinese Academy of Sciences, Huzhou, China; 3 Hunan Engineering Research Center of Combinatorial Biosynthesis and Natural Product Drug Discovery, Changsha, Hunan, China; 4 Xiangya International Academy of Translational Medicine, Central South University, Changsha, Hunan, China; Auburn University, United States of America

## Abstract

Erythromycins (Ers) are clinically potent macrolide antibiotics in treating pathogenic bacterial infections. Microorganisms capable of producing Ers, represented by *Saccharopolyspora erythraea*, are mainly soil-dwelling actinomycetes. So far, *Actinopolyspora erythraea* YIM90600, a halophilic actinomycete isolated from Baicheng salt field, is the only known Er-producing extremophile. In this study, we have reported the draft genome sequence of *Ac. erythraea* YIM90600, genome mining of which has revealed a new Er biosynthetic gene cluster encoding several novel Er metabolites. This Er gene cluster shares high identity and similarity with the one of *Sa. erythraea* NRRL2338, except for two absent genes, *eryBI* and *eryG*. By correlating genotype and chemotype, the biosynthetic pathways of 3′-demethyl-erythromycin C, erythronolide H (EH) and erythronolide I have been proposed. The formation of EH is supposed to be sequentially biosynthesized via C-6/C-18 epoxidation and C-14 hydroxylation from 6-deoxyerythronolide B. Although an *in vitro* enzymatic activity assay has provided limited evidence for the involvement of the cytochrome P450 oxidase EryF^Ac^ (derived from *Ac. erythraea* YIM90600) in the catalysis of a two-step oxidation, resulting in an epoxy moiety, the attempt to construct an EH-producing *Sa. erythraea* mutant via gene complementation was not successful. Characterization of EryK^Ac^ (derived from *Ac. erythraea* YIM90600) *in vitro* has confirmed its unique role as a C-12 hydroxylase, rather than a C-14 hydroxylase of the erythronolide. Genomic characterization of the halophile *Ac. erythraea* YIM90600 will assist us to explore the great potential of extremophiles, and promote the understanding of EH formation, which will shed new insights into the biosynthesis of Er metabolites.

## Introduction

Erythromycins (Ers) are a series of 14-membered macrolide antibiotics showing broad-spectrum activity against various gram-positive bacteria [1]. Since its first discovery in *Saccharopolyspora erythraea* (*Sa. erythraea*, formerly known as *Streptomyces erythraeus*), Ers have been reported clinically potent in treating certain types of pathogenic bacterial infections [2], [3]. However, natural Ers are facilely decomposable under acidic conditions, which will result in the loss of clinic activity and the appearance of undesirable side effects [4]. To minimize acidic instability, second-generation Ers such as clarithromycin [5], azithromycin [6], and roxithromycin [7] with modified macrolide skeletons were generated. Nowadays, the rising bacterial resistance encountered by second-generation Ers becomes noticeable. The possible resistance mechanism may come from ribosomal modification (erm) or macrolide efflux (mef) [8], [9]. In either way, the drug activity will be reduced dramatically. Therefore, the development of third-generation Ers, represented by cethromycin (ABT-773) [10], EP-420 [11], and BAL-19403 [12], was addressed to overcome the bacterial resistance problem.

**Figure 1:**
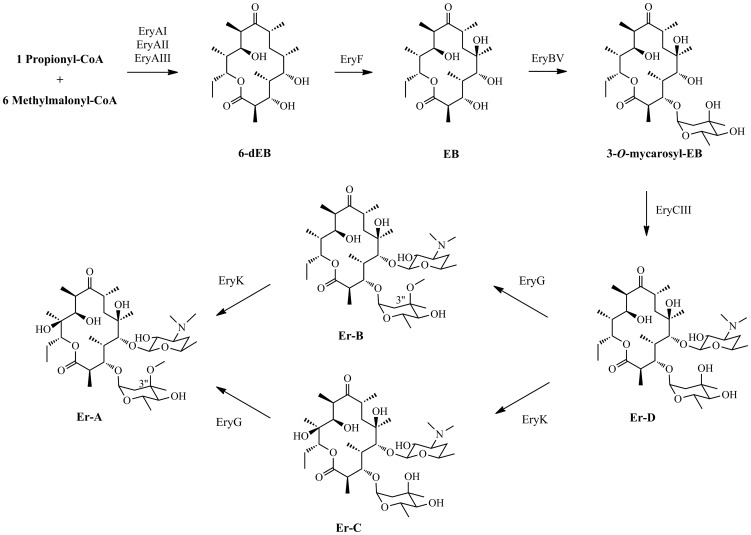
Biosynthetic pathway of the final Er product, Er-A. Three DEBSs, EryAI-AIII, are responsible for the generation of the 16-membered lactone, 6-dEB; Tailoring enzymes catalyze sequential reactions, including two hydroxylations, two glycosylations, and one methylation, to obtain the final product, Er-A.

As a model organism for laboratory researches and a parental strain for industrial production, *Sa. erythraea* has been extensively and systemically investigated. The erythromycin biosynthetic gene cluster in *Sa. erythraea* NRRL2338 has been characterized and validated by the genome sequence-based analysis [13], [14]; while erythromycin A (Er-A), B (Er-B), and C (Er-C) have also been classified as its major products via numerous fermentative analyses [15]–[17]. Correlation between genotype and chemotype has facilitated us to understand the biosynthesis of Ers in *Sa. erythraea*: the assembling of the 6-deoxyerythronolide B (6-dEB) skeleton from one propionyl-coenzyme A (CoA) and six methylmalonyl-CoAs by a set of multifunctional type I polyketide synthases (PKSs) named 6-deoxyerythronolide B synthases (DEBSs) [18], [19], and the sequential post-PKS modifications, including two hydroxylations, two glycosylations, and one methylation, to form the final product, Er-A [20] (Fig. 1).

**Figure 2:**
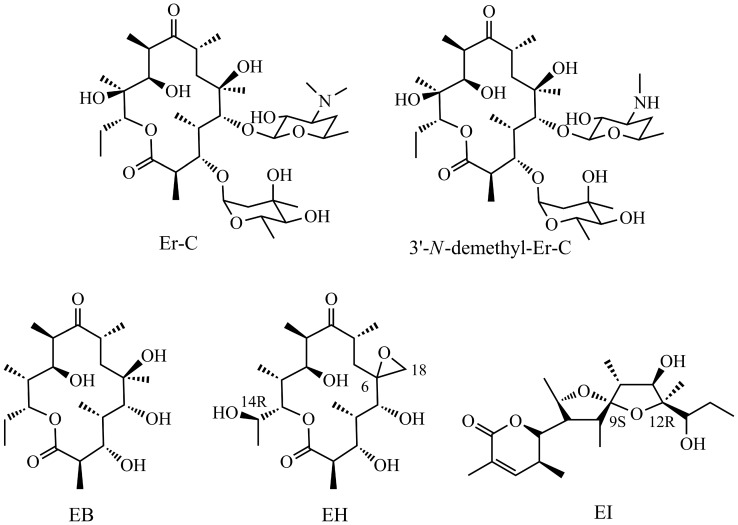
The identified Er metabolites in *Ac. erythraea* YIM90600. EB and Er-C are normal Er intermediates, while 3′-*N*-demethyl-Er-C, EH, and EI are novel Er congeners that have rarely been reported.

So far, the reported microorganisms capable of producing Ers are mainly soil actinomycetes. In addition to *Sa. erythraea*, Ers have also been detected in certain strains of *Arthrobacter* and *Nocardia* species [21], [22]. Comparing with those soil-dwelling microbes, the original Er-producing strain *Actinopolyspora erythraea* (*Ac. erythraea*) YIM90600 in this study was isolated from Baicheng salt field in Xingjiang province, northwestern of China [23]. As the representatives of halophilic species, *Actinopolyspora* species normally require high salinity environment for growth. The metabolites analyses of *Ac. erythraea* YIM90600 from large-scale fermentation cultures have confirmed the presence of several normal Er biosynthetic intermediates and novel congeners, along with three linear polyketide actinopolysporin A, B, C and a known antineoplastic antibiotic tubercidin [24]. Specifically, the identified Er metabolites in YIM90600 include Er-C, 3′-*N*-demethyl-Er-C, erythronolide B (EB), erythronolide H (EH) and erythronolide I (EI) [25] (Fig. 2), which hints the possible existence of an Er-like gene cluster in the genome of YIM90600. Meanwhile, the presence of novel erythronolides, EH and EI, may indicate some distinct biosynthetic pathways in YIM90600. Moreover, EH exhibites identical chemical structure to EB except the C-6/C-18 epoxidation and the C-14 hydroxylation, thus it could be served as an ideal aglycone for further chemical or enzymatic modification [25].

In this work, we have taken advantage of whole-genome sequencing and computer-assisted analysis to identify and characterize the Er biosynthetic gene cluster in *Ac. erythraea* YIM90600. The established genotype-chemotype correlation could facilitate us to decipher the possible formation of Er congeners in YIM90600 and may shed new insights into the Er biosynthetic pathway, as well as the development of novel Er derivatives.

## Materials and Methods

### Bacterial strains, plasmids, and reagents

The strains and plasmids used in this study were summarized in Table 1. Primer sequences were listed in Table S1 in File S1. Chemicals and biochemical materials were purchased from standard commercial sources.

**Table 1:** Bacterial strains and plasmids used and constructed in this study.

*Strains/plasmids*	*Characteristic(s)*	*Source/reference*
*E. coli*		
DH5α	Host for general cloning	Invitrogen
ET12567(pUZ8002)	Donor strain for conjugation between *E. coli* and *Streptomyces*	[26]
BL21(DE3)	Host for protein expression	Novagen
EX104	Derivative of BL21(DE3) containing an EryF^Sa^-expression vector	This study
EX105	Derivative of BL21(DE3) containing an EryF^Ac^-expression vector	This study
EX106	Derivative of BL21(DE3) containing an EryK^Sa^-expression vector	This study
EX107	Derivative of BL21(DE3) containing an EryK^Ac^-expression vector	This study
*Ac. erythraea*		
YIM90600	Original Er-producing strain, a halophilic actinomycete from slat field	[23]
*Sa. erythraea*		
ZL2001	Recombinant Er-producing strain, enhancing the expression of *eryK* and *eryG*, introducing artificial *attB* sites for site-specific recombination	[15], [16]
EX101	Derivative of ZL2001, *eryF^Sa^* in-frame deletion mutant	This study
EX102	Derivative of ZL2001, *eryBV^Sa^* in-frame deletion mutant	This study
EX103	Derivative of EX101 containing pEX103 integrated by the ΦC31 integrase-mediated recombination with the genotype of PermE*-eryF^Ac^	This study
Plasmids		
pMD18-T simple	*E. coli* subcloning vector	TaKaRa
pET28a	*E. coli* protein expression vector	Novagen
pOJ260	*E. coli-Streptomyces* shuttle vector containing the *aac(3)IV* gene	[43]
pSET152	*E. coli-Streptomyces* shuttle vector containing *aac(3)IV* gene, the ΦC31 *attP* site and integrase gene	[43]
pWHM79	pGEM3zf derivative carrying a 0.5-kb fragment containing the ermE* promotor	[42]
pEX1011	pMD19-T derivative carrying the upstream fragment of *eryF^Sa^*	This study
pEX1012	pMD19-T derivative carrying the downstream fragment of *eryF^Sa^*	This study
pEX1021	pMD19-T derivative carrying the upstream fragment of *eryBV^Sa^*	This study
pEX1022	pMD19-T derivative carrying the downstream fragment of *eryBV^Sa^*	This study
pEX101	pOJ260 derivative for in-frame deletion within *eryF^Sa^*	This study
pEX102	pOJ260 derivative for in-frame deletion within *eryBV^Sa^*	This study
pEX1031	pMD19-T derivative carrying the *eryF^Ac^* gene fragment with its terminator	This study
pEX103	pSET152 derivative for the PermE*-controlled expression of *eryF^Ac^*	This study
pEX1041	pMD19-T derivative carrying the *eryF^Sa^* gene	This study
pEX104	pET28a derivative for heterologous expression of EryF^Sa^	This study
pEX1051	pMD19-T derivative carrying the *eryF^Ac^* gene	This study
pEX105	pET28a derivative for heterologous expression of EryF^Ac^	This study
pEX1061	pMD19-T derivative carrying the *eryK^Sa^* gene	This study
pEX106	pET28a derivative for heterologous expression of EryK^Sa^	This study
pEX1071	pMD19-T derivative carrying the *eryK^Ac^* gene	This study
pEX107	pET28a derivative for heterologous expression of EryK^Ac^	This study

### DNA isolation, manipulation, and sequencing

DNA isolation and manipulation in *Escherichia coli* (*E. coli*) and *Sa. erythraea* were performed according to standard salting-out protocol [26]. The procedure for extraction and purification of *Ac. erythraea* genomic DNA was modified by replacing the lysozyme digestion with liquid-nitrogen grinding. Primer synthesis and DNA sequencing were performed by Shanghai GeneCore Biotechnology Inc. Whole-genome sequencing of *Ac. erythraea* YIM90600 was performed by Beijing Genomics Institute (BGI)-Shenzhen. Draft genome sequence of *Ac. erythraea* YIM90600 was deposited at DDBJ/EMBL/GenBank with the accession number JPMV00000000. DNA sequences covering left, middle, and right fringes of the Er gene cluster were deposited in GenBank with accession numbers KJ143518, KJ143519, and KJ143520, respectively.

### Gene disruption of *eryF^Sa^* and *eryBV^Sa^* in *Sa. erythraea* ZL2001

The genomic DNA of *Sa. erythraea* ZL2001 was served as the template for PCR amplification.

To inactivate EryF^Sa^ in the original strain ZL2001, a 2.02 kb fragment amplified by primers pFLf and pFLr and a 2.13 kb fragment amplified by primers pFRf and pFRr were initially cloned into the pMD19-T vector, giving pEX1011 and pEX1012, respectively. After DNA sequencing to confirm the fidelity, the 2.02 kb *Eco*RI/*Xba*I and 2.13 kb *Xba*I/*Hin*dIII fragments were recovered and then co-ligated into the *Eco*RI/*Hin*dIII site of pOJ260, yielding the recombinant plasmid pEX101, in which a 771 bp in-frame coding region (corresponding to AA_71_-AA_327_ of the deduced product EryF^Sa^) of *eryF^ Sa^* was deleted.

To inactivate EryBV^Sa^ in the original strain ZL2001, a 2.04 kb fragment amplified by primers pBVLf and pBVLr and a 2.08 kb fragment amplified by primers pBVRf and pBVRr were initially cloned into the pMD19-T vector, giving pEX1021 and pEX1022, respectively. After DNA sequencing to confirm the fidelity, a recombinant plasmid pEX102 was constructed following the same strategy, in which a 786 bp in-frame coding region (corresponding to AA_76_-AA_337_ of the deduced product EryBV^Sa^) of *eryBV^Sa^* was deleted.

The constructs pEX101 and pEX102 were introduced individually into *Sa. erythraea* ZL2001 by intergeneric conjugation from *E. coli* ET12567/pUZ8002. Following the procedure described previously [15], the exconjugants were subjected to a double-crossover recombination event, leading to the generation of mutant strains EX101 and EX102, respectively. The genotype of each mutant was validated by PCR amplification (Figure S1 and Figure S2 in File S1).

### Gene complementation of *eryF^Ac^* in *Sa. erythraea* EX101

The genomic DNA of *Ac. erythraea* YIM90600 was served as the template for PCR amplification.

To complement *eryF^Ac^* into the *eryF^Sa^*-deleting mutant EX101, a 1.29 kb fragment obtained by using the primers pAcF-Cf and pAcF-Cr was initially cloned into the pMD19-T vector, giving pEX1031. After sequencing to confirm the fidelity, the 1.29 kb *Hin*dIII/*Xba*I fragment and a 0.49 kb *Eco*RI/*Hin*dIII fragment containing PermE* were recovered and co-ligated into the *Eco*RI/*Xba*I site of pSET152 to yield pEX103.

The construct pEX103 was introduced into *Sa. erythraea* EX101 by intergeneric conjugation from *E. coli* ET12567/pUZ8002, following the procedure described previously [16]. The colonies presenting apramycin-resistant phenotype were identified as exconjugants, leading to the generation of the recombinant strains EX103. The genotype of the complementary strain was confirmed by PCR amplification-coupled sequencing described previously [16], [27] (Figure S3 in File S1).

### Fermentation and chemical analyses of the Er metabolites

Cultivation of the *Sa. erythraea* strains and compound extraction from the fermentative broths were carried out according to the procedures described previously [16]. High performance liquid chromatography-electrospray ionization-mass spectrometry (HPLC-ESI-MS) analysis was performed on a Shimadzu 2010 liquid chromatograph-mass spectrometer (Shimadzu, Japan), and a Diamonsil C18 5 µ reverse-phase column (250×4.6 mm; catalog no. 99603; Dikma, USA). The analytical method was developed with a flow rate of 1 ml/min and column temperature at 23 °C. The column was eluted using an 35 min gradient program: 0–3 min, constant 85% A/15% B; 3–6 min, a linear gradient to 60% A/40% B; 6–12 min, constant 60% A/40% B; 12–19 min, a linear gradient to 45% A/55% B; 19–22 min, a linear gradient to 15% A/85% B; and 22–35 min, constant 15% A/85% B (solvent A, 5 mM NH_4_COOH, 0.5‰ HCOOH in H_2_O; solvent B, 0.5‰ HCOOH in CH_3_CN). High-resolution ESI-MS (HR-ESI-MS) analysis was carried out on a maXis 4G ultra-high resolution time-of-flight (UHR-TOF) mass spectrometer (Bruker Daltonics, USA). ESI-MS-MS analysis was carried out on an LTQ Orbitrap XL mass spectrometer (Thermo Fisher Scientific, USA).

### Expression and purification of EryF^Sa^, EryF^Ac^, EryK^Sa^ and EryK^Ac^

A 1.22 kb *eryF^Sa^* gene fragment and a 1.20 kb *eryK^Sa^* gene fragment were amplified from the genomic DNA of *Sa. erythraea* ZL2001 using primer pairs pSaFf/pSaFr and pSaKf/pSaKr, respectively. The PCR products were individually cloned into the pMD19-T vector. After sequencing to confirm the fidelity, the inserts were recovered and ligated into the *Nde*I/*Eco*RI site of pET28a, yielding pEX104 and pEX106 for expression of *N*-terminal 6 x His-tagged EryF^Sa^ and EryK^Sa^, respectively. Similarly, a 1.22 kb *eryK^Sa^* gene fragment and a 1.20 kb *eryK^Ac^* gene fragment were amplified from the genomic DNA of *Ac. erythraea* YIM90600 using primer pairs pAcFf/pAcFr and pAcKf/pAcKr, respectively. Following the same sub-cloning steps, pEX105 and pEX107 were constructed for producing *N*-terminal 6 x His-tagged EryF^Ac^ and EryK^Ac^, respectively.

The constructs pEX104, pEX105, pEX106 and pEX107 were introduced individually into *E. coli* BL21 (DE3) by transformation, yielding four recombinant strains EX104, EX105, EX106 and EX107, respectively. Each strain was cultured in Luria Bertani (LB) medium supplemented with 50 µg/ml kanamycin at 37 °C and 250 rpm until OD_600_ reached 0.6–0.8. To induce protein expression, 0.1 mM isopropyl-β-D-thiogalactopyranoside (IPTG), 0.1 mM Fe(NH_4_)_2_(SO_4_)_2_, and 0.5 mM 5-aminolevulinic acid (ALA) were added to the culture before further incubation at 18 °C and 250 rpm for 28–32 hrs. Purification and quantification of the targeted proteins were carried out according to the methods described previously [28] (Figure S4 in File S1).

### Characterization of EryF^Sa^, Ery^Ac^, EryK^Sa^ and EryK^Ac^

To characterize EryF^Sa^, Ery^Ac^, EryK^Sa^ and EryK^Ac^ as cytochrome P450 proteins, the CO binding difference spectra within the range of 350–500 nm, were recorded as previously described [28] (Figure S5 in File S1). Following the procedure described before, substrate binding spectra were recorded after the addition of 6-dEB dissolved in dimethyl sulfoxide (DMSO) [28] (Figure S9 in File S1). Measurement of ultraviolet-visible (UV-vis) absorbance was performed on a JASCO V-530 UV/vis spectrophotometer (Jasco, Japan).

A 50 µl reaction mixture in 10% (v/v) DMSO containing 50 µM 6-dEB, 1 mM NADPH, 0.1 U/ml ferredoxin-NADP^+^ reductase, 50 µg/ml ferredoxin, 10 mM glucose-6-P, 1 U/ml glucose-6-P dehydrogenase and 2 µM EryF^Sa^ (or EryF^Ac^) in 50 mM Tris-HCl buffer (pH 7.5) was incubated at 30°C for 2 hrs. For a negative control, EryF^Sa^ (or EryF^Ac^) was inactivated by heating at 100°C for 15 min. Similarly, 0.2 mM Er-B, was incubated with 2 µM EryK^Sa^ (or EryK^Ac^) (other components were the same as the reaction mixture mentioned above). For a negative control, EryK^Sa^ (or EryK^Ac^) was inactivated by heating at 100°C for 15 min. Reactions were initiated by addition of target enzyme, and terminated by adding an equal volume of acetonitrile to precipitate the enzyme. After removal of the precipitate by centrifugation, the supernatant was subjected to HPLC-ESI-MS, HR-ESI-MS and ESI-MS-MS analyses.

## Results and Discussion

### Genomic characterization of the halophilic actinomycete *Ac. erythraea* YIM90600

Halophiles are conveniently grouped according to their physiological requirement for salinity environment. Slight halophiles, mainly isolated from marine, favor a living condition of 2–5 % NaCl. Moderate halophiles prefer a wider NaCl concentration range of 5–20 %. Extreme halophiles, represented by the acknowledged halobacteria and halococci, grow well at NaCl concentrations higher than 20 % [29]. In this study, the original strain *Ac. erythraea* YIM90600, first isolated from Baicheng salt field of China, is a typical moderate halophile [23]. By using ISP4 (International *Streptomyces* Project 4) agar media supplemented with different concentrations of NaCl, we have validated that YIM90600 favors an environment of 10–20 % NaCl. Meanwhile, YIM90600 is able to grow at NaCl concentrations higher than 25 %, but not in low salinity environment. According to previous literatures on taxonomy, the genus *Actinopolyspora*, belonging to the suborder *Acintopolysporineae*, includes high G+C Gram-positive bacteria [30].

The genomic DNA of *Ac. erythraea* YIM90600 was extracted following a modified salting-out method from a 4-day TSB (Tryptic Soy Broth) culture supplemented with 15 % NaCl. The resultant DNA sample, showing a mean length of about 25 kb and OD_260_/OD_280_ of 1.78, was qualified for further genomic sequencing. Whole-genome sequencing of YIM90600 was performed with a strategy of Illumina paired-end sequencing technology [31]. About 960 Mb raw data of a 500bpPCR-free index library and about 518 Mb raw data of a 2000bpPCR-free index library were generated. Clean data filtered from both libraries were assembled into 37 scaffolds and 86 contigs. De novo assembly yielded a 5.36 Mb draft genome sequence with a mean G+C content of 68.76 %. Sequence similarity searching for known proteins was conducted in the COG (Clusters of Orthologous Groups) database, the Swiss-Prot protein database, and the KEGG (Kyoto Encyclopedia of Genes and Genomes Pathway) database. A total of 5303 genes covering 84.39 % of the genome were annotated for their function. The average gene length was determined as 852 bp. Searching against the KEGG database indicated that 2293 genes were mapped to 170 KEGG pathways [32]. Additionally, 102 genes involved in metabolism of terpenoids and polyketides were identified, which suggested that they were most likely responsible for the biosynthesis of Er metabolites. Genomic characterization of the halophilic actinomycete *Ac. erythraea* YIM90600 would help us to explore its potential for the production of secondary metabolites, and the biosynthetic mechanisms of YIM90600-derived natural products.

### Identification and preliminary analysis of the Er gene cluster in *Ac. erythraea* YIM90600

**Figure 3:**
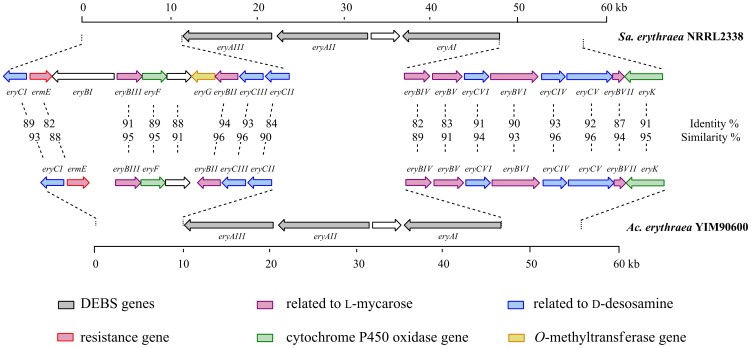
Comparative analysis of the two gene clusters of *Sa. erythraea* NRRL2338 and *Ac. erythraea* YIM90600. The genes share high identity (82–93 %) and similarity (88–96 %). Most of the genes exhibit the same order and direction as their homologues, except for *eryBI* and *eryG*, which are absent in the newly identified gene cluster.

Genome mining of *Ac. erythraea* YIM90600 enabled us to explore its genetic basis for Er production. By searching the 5.36 Mb draft genome sequence for *debs* homologues, we have identified three separated fragments which could be merged to form a full-length Er gene cluster in YIM90600. The newly identified ORFs (Open Reading Frames) were named following their homologous genes in *Sa. erythraea* (Fig. 3). To avoid confusion, two superscripts, *Sa* and *Ac*, were used to distinguish genes from *Sa. erythraea* and *Ac. erythraea*, respectively.

The Er-encoding genes in YIM90600 shares high identity (82–93 %) and similarity (88–96 %) with their homologues in *Sa. erythraea* NRRL2338 [13], [14], suggesting that their transcriptional products present similar functions. A series (EryAI-AIII) are PKS enzymes responsible for the assembling of the macrolactone, 6-dEB [18], [19]; B series (EryBII-BVII) and C series (EryCI-CVI) are enzymes related to the formation and attachment of the sugar residues, L-mycarose and D-desosamine, respectively [33], [34]; Tailoring enzymes, including cytochrome P450 oxidases (EryF and EryK) and glycosyltransferases (EryBV and EryCIII), catalyze the post-PKS modifications of polyketide skeleton [20] (Fig. 1). With comparison, the Er-encoding genes in both strains exhibited the same order and direction, except that *eryBI* and *eryG* are absent in YIM90600 (Fig. 3). Interestingly, there have been different opinions on the biochemical function of EryBI. The amino acid sequence suggests that EryBI belongs to β-glucosidase, indicating its involvement in bioconversion of sugar residues [35]. Based on its B series nomenclature, EryBI should be involved in L-mycarose bioconversion [14]. From the perspective of homology, EryBI also showed 61% identity and 74 % similarity to OleR in oleandomycin biosynthesis [36], suggesting it might play an important role as resistant protein. However, neither of these viewpoints is tenable since the inactivation of EryBI exerted no effect on the Er production in *Sa. erythraea* [37]. Therefore, *eryBI* could be a nonfunctional gene, and this assumption has been further confirmed by our genomic analyses results that the discovered Er-producing machinery in YIM90600 does not contain any *eryBI* homologue. Comparative analysis of the two Er gene clusters would allow us to study the biosynthetic mechanisms of Er metabolites in YIM90600, further characterization of which would also provide insights into the generality in Er formation, as well as the specificity in EH and EI biosyntheses.

### Genotype-chemotype correlation of Er metabolites in *Ac. erythraea* YIM90600

The distinct Er metabolites profile of *Ac. erythraea* YIM90600 has presented a certain consistency to its genotype [25]. The absence of normal main Er compounds such as Er-A and Er-B in YIM90600 is probably due to the absence of *eryG*, a gene encoding a methyltransferase responsible for the *O*-methylation at C’’-3 of the mycarosyl residue [38], thus the intermediate Er-C is accumulated. Based on the biosynthetic pathway, the P450 oxidase EryF is responsible for the C-6 hydroxylation of 6-dEB, and the resultant EB is the first intermediate after the tailoring steps [20], [39]. The disappearance of EB in *Sa. erythraea* may because of the fast and efficient subsequent modification step of C-3 *O*-glycosylation; while the accumulation of EB in YIM90600 suggests that its glycosylation step is relatively not sufficient, probabaly due to either inefficiency of the glycosyltransferase activity or inadequacy of the sugar supply. 3′-*N*-demethyl-Er-C differs from Er-C in the *N*-methylation degree at C′-3 of the desosaminyl residue. According to that, EryCVI responsible for the *N*-dimethylation step during desosamine biosynthesis [33], [34] may only exert its partial activity in YIM90600, which could lead to the generation of monomethylated product.

**Figure 4:**
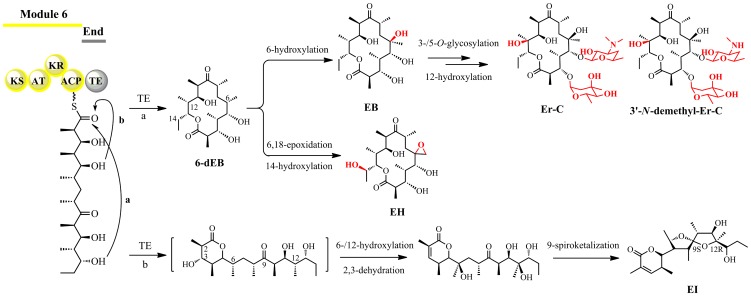
Proposed biosynthetic pathways of the Er metabolites in *Ac. erythraea* YIM90600 modeled on erythromycin biosynthesis in *Sa. erythraea* [25]. The EryAIII TE domain is supposed dual functional. The TE-catalyzed intramolecular cyclization releases the 14-membered 6-dEB via path a, and a 6-membered lactone via path b. Both serve as substrates for further modifications.

The biosynthetic pathways of novel EH and EI have been proposed before [25] (Fig. 4). The DEBS thioesterase (TE) domain in YIM90600 was predicted dual functional in catalyzing two different intramolecular cyclizations, resulting in both 14- and 6-membered lactones. The sequential C-6/C-18 epoxidation and C-14 hydroxylation of the 14-membered lactone 6-dEB could generate EH; while the 6-membered lactone might undergo C-6 and C-12 hydroxylations, C-2/C-3 dehydration, and C-9 spiroketalization to form EI. Appearance of the shunt metabolite EH hinted us the existence of certain cytochrome P450 oxidase(s) in catalyzing the typical epoxy moiety and the additional hydroxyl group.

### Characterization of the cytochrome P450 protein EryF^Ac^ via *in vitro* enzymatic activity assay and *in vivo* gene complementation

Base on our hypothesis, EryF, acting as a C-6 hydroxylase for 6-dEB in *Sa. erythraea*, may catalyze a successive two-step oxidation in *Ac. erythraea*, which coverts 6-dEB into 6, 18-epoxy-EB via the intermediate EB. To evaluate whether EryF^Ac^ is capable of catalyzing a four-electron oxidation, a corresponding *in vitro* enzymatic activity assay of the P450 oxidase has been conducted. The *N*-6 x His-tagged recombinant proteins, EryF^Sa^ and EryF^Ac^, were respectively expressed, purified, and characterized as cytochrome P450 proteins (Figure S4 and Figure S5 in File S1) [28].

**Figure 5:**
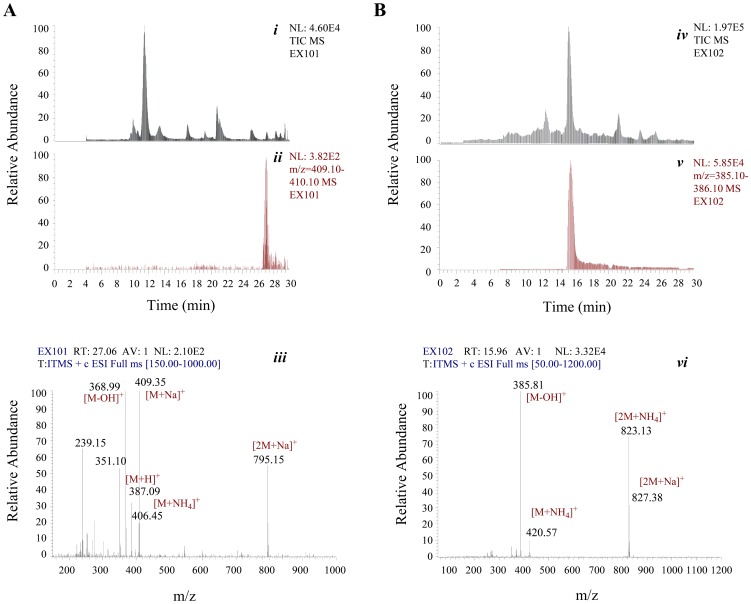
HPLC-ESI-MS analyses of the fermentation cultures of *Sa. erythraea* EX101 and EX102. (A) Total ion current chromatogram (*i*), and reconstructed base peak chromatogram for 6-dEB (*ii*) of the fermentation products of EX101. ESI-MS data recorded at the retention time of 27.06 min (*iii*). (B) Total ion current chromatogram (*iv*), and reconstructed base peak chromatogram for EB (*v*) of the fermentation products of EX102. ESI-MS data recorded at the retention time of 15.96 min (*vi*).

*Sa. erythraea* ZL2001 is a recombinant strain with introduction of 8 copies of *attB* (attachment of bacterial) sites in its genome to enhance actinophage ΦC31 integrase-mediated site-specific recombination [15], [16], and possesses the overexpression of EryK and EryG to strengthen the production of the most potent component, Er-A (Figure S10 in File S1). In-frame deletion of *eryF^Sa^* in ZL2001 generated a mutant *Sa. erythraea* EX101, which has completely abrogated the virulence of natural Er components, validating the indispensability of EryF^Sa^. The fermentation of EX101 led to the accumulation of 6-dEB, the substrate for *in vitro* enzymatic activity assay, and the generation of novel 6-deoxy-Ers (Fig. 5A, and Figure S11 in File S1). The appearance of 6-deoxy-Ers suggested that inactivation of EryF^Sa^ could not completely block the subsequent Er tailoring steps in *Sa. erythraea*. The identity of 6-dEB was confirmed by HR-ESI-MS and ESI-MS-MS analyses (Figure S6A and Figure S7 in File S1). Meanwhile, the one-step oxidation product EB was produced in an *eryBV^Sa^*-deleting mutant, *Sa. erythraea* EX102 (Fig. 5B). In consistence, in-frame deletion of *eryBV^Sa^* led to the detection of Er metabolites with only desosaminyl residue (Figure S12 in File S1), further indicating the tolerance of tailoring enzymes. HR-ESI-MS and ESI-MS-MS analyses were carried out to accomplish the identity of EB (Figure S6B and Figure S8 in File S1).

**Figure 6:**
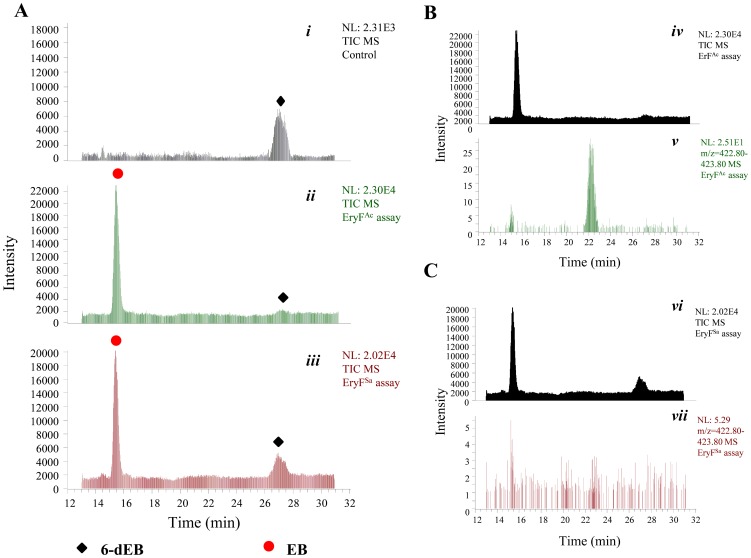
HPLC-ESI-MS analyses of the *in vitro* enzymatic reactions catalyzed by EryF^Sa^ and EryF^Ac^, respectively. (A) Total ion current chromatograms indicating the *in vitro* conversion of 6-dEB to EB in the absence of active EryF^Ac^ (*i*), in the presence of active EryF^Ac^ (*ii*), or in the presence of active EryF^Sa^ (*iii*). (B) Total ion current chromatogram (*iv*) and reconstructed base peak chromatogram for 6, 18-epoxy-EB (*v*) of the EryF^Ac^ reaction mixture. (C) Total ion current chromatogram (*vi*) and reconstructed base peak chromatogram for 6, 18-epoxy-EB (*vii*) of the EryF^Sa^ reaction mixture.

The *in vitro* assays using 6-dEB and EryF^Sa^ (or EryF^Ac^) were carried out. In the presence of EryF^Ac^, 6-dEB was consumed accordingly to generate EB, as well as a possible new product, showing its positive ion peak at m/z 423.3. In the presence of EryF^Sa^, no apparent ion peak corresponding to such new product was detected (Fig. 6). Further HR-ESI-MS analysis has eventually established the molecular formula of this new product as C_21_H_36_O_7_ ([M+Na]^+^ m/z calculated 423.2353, found 423.2342), suggesting its identity to 6, 18-epoxy-EB, which differs from EB with an distinct epoxide group (Figure S6C in File S1).

The original EH-producing strain *Ac. erythraea* YIM90600 is not suitable for large-scale fermentation, because of its long cell growth cycle and harsh fermentative condition, as well as its low titers of Er metabolites [23], [25]. On the contrary, the genetic recombinant strain *Sa. erythraea* ZL2001, showing favorable physiological properties and high Er productivity, can be served as an ideal host for EH production [15]. In addition, the artificial *attB* sites in ZL2001 are quite convenient for genetic manipulation [16].

**Figure 7:**
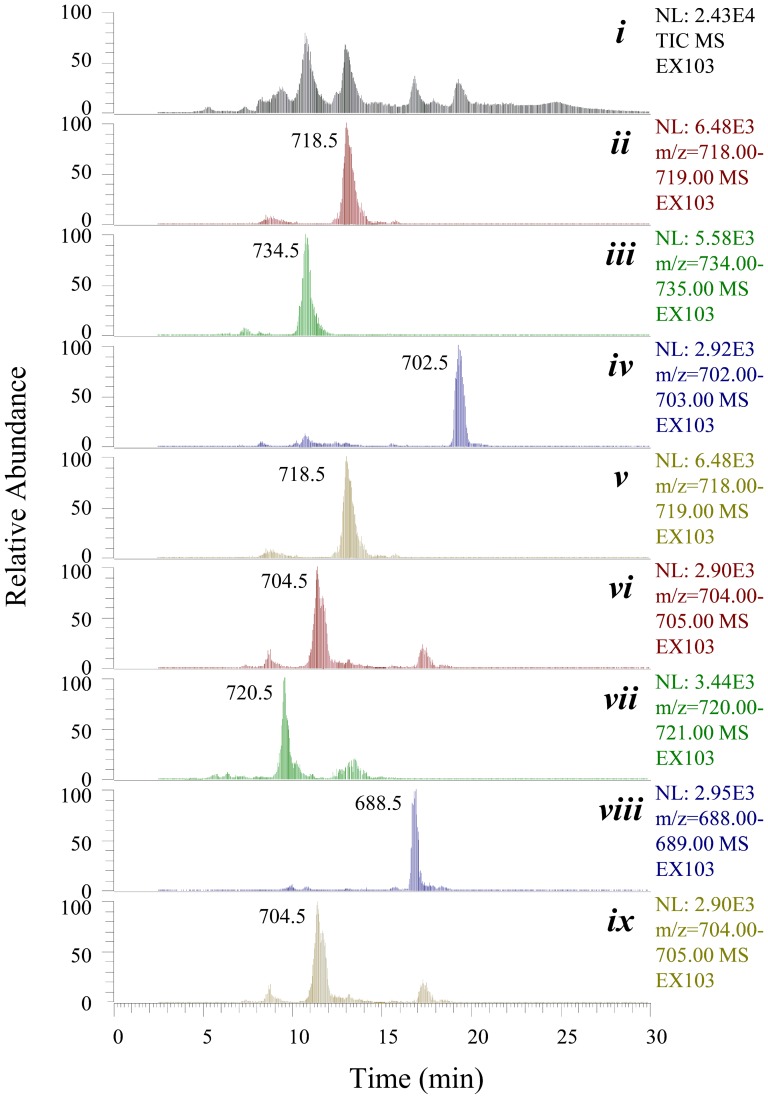
HPLC-ESI-MS analysis of the fermentation culture of *Sa. erythraea* EX103. Total ion current chromatogram (*i*), and reconstructed base peak chromatograms for 6-deoxy-Er-A (*ii*), Er-A (*iii*), 6-deoxy-Er-B (*iv*), Er-B (*v*), 6-deoxy-Er-C (*vi*), Er-C (*vii*), 6-deoxy-Er-D (*viii*), and Er-D (*ix*) are recorded. Note that 6-deoxy-Er-A and Er-B, as well as 6-deoxy-Er-C and Er-D share the same molecular weights and similar polarities, their base peaks are thus overlapping.

Inspired by the previous success in generating new Er analogues via heterologous expression of OleP, a cytochrome P450 oxidase catalyzing an epoxidation of the oleandomycin lactone ring [40], [41], we have attempted to investigate whether *in vivo* gene complementation of *eryF^Ac^* into the *eryF^Sa^*-deleting strain would lead to the accumulation of EH in such mutant. The pEX103, a pSET152-derived plasmid containing *eryF^Ac^* gene fragment under the control of a potent promoter P_ermE_* [42], was introduced into EX101 to yield the recombinant strain *Sa. erythraea* EX103 (Figure S3 in File S1). Its genotype was validated as *attL*-linear pEX103-*attR* [16], [27]. EX103 has restored the production of major Er components including Er-A, Er-B, and Er-C (Fig. 7), confirming the successful introduction of the target gene fragment P_ermE_*- *eryF^Ac^* at the artificial *attB* site, as well as the association of such new gene to continue the biosynthesis of Ers. However, neither EH nor 6, 18-epoxy-EB was detected in the fermentation cultures of EX103 (Figure S13 in File S1).

According to our analysis, the unique two-step oxidation catalyzed by EryF^Ac^ in the halophile *Ac. erythraea* YIM90600 may count on the insufficient conversion of EB to 3-*O*-mycarosyl-EB. Thus, the redundant EB could induce the occurrence of second oxidation catalyzed by EryF^Ac^ and the formation of 6,18-epoxy-EB, which finally leads to the production of a small amount of EH. Therefore, the invalidation of EryF^Ac^ to catalyze a successive two-step oxidation in *Sa. erythraea* mutant may due to the limited acquisition of EB, which is quite efficiently converted to 3-*O*-mycarosyl-EB. Further genetic manipulation targeting on EryBV shall provide us more clues to decipher such divergence.

### Characterization of the cytochrome P450 protein EryK^Ac^ via *in vitro* enzymatic activity assay

The additional hydroxyl group of EH at C-14 could be catalyzed by a regiospecific P450 oxidase. The C-12 hydroxylation of Er-D (or Er-B) in *Sa. erythraea* is catalyzed by EryK, hinting a possibility of its additional function as a C-14 hydroxylase in *Ac. erythraea* to form EH. Two recombinant P450 proteins, EryK^Sa^ and EryK^Ac^, were expressed, purified and characterized respectively (Figure S4 and Figure S5 in File S1).

**Figure 8:**
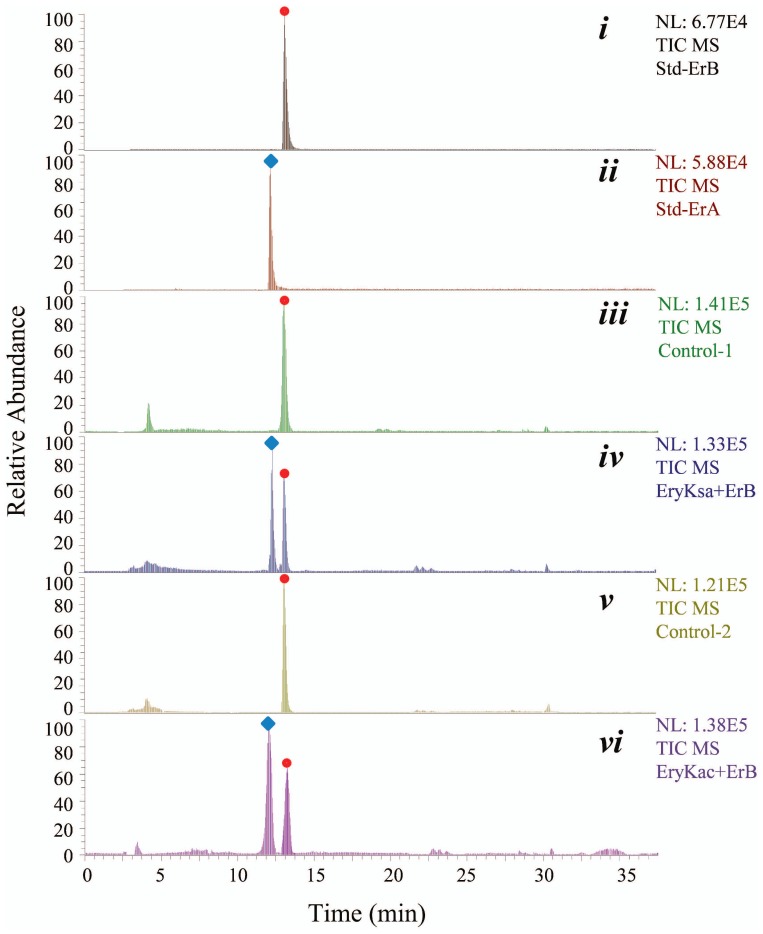
HPLC-ESI-MS analyses of the *in vitro* enzymatic reactions catalyzed by EryK^Sa^ and EryK^Ac^, respectively. Total ion current chromatograms indicating standard Er-B (red circle, *i*) and Er-A (blue lozenge, *ii*), the *in vitro* conversion of Er-B to Er-A in the absence of active EryK^Sa^ (*iii*), in the presence of active EryK^Sa^ (*iv*), in the absence of active EryK^Ac^ (*v*), or in the presence of active EryK^Ac^ (*vi*).

Using standard Er-B as the substrate, the enzymatic activities of EryK^Sa^ and EryK^Ac^ were tested *in vitro*. In the presence of EryK^Sa^ (or EryK^Ac^), Er-B was consumed accordingly with the generation of Er-A (Fig. 8), confirming that the proteins were in the active form and the function of EryK^Ac^ as a C-12 hydroxylase in generating Er-C and 3′-*N*-demethyl-Er-C in *Ac. erythraea*. And then, 6-dEB was incubated with both EryF^Sa^ and EryK^Sa^ (or with both EryF^Ac^ and EryK^Ac^). However, no apparent ion peak corresponding to 12-hydroxyl-EB or EH was detected (Figure S14 in File S1).

## Conclusions

Based on genome sequencing and bioinformatics analysis, we have identified a new Er biosynthetic gene cluster in a halophilic actinomycete *Ac. erythraea* YIM90600. This Er gene cluster shares high identity and similarity with the one of *Sa. erythraea* [13], [14], but lacks two genes *eryBI* and *eryG*. Correlation of genotype and chemotype has increased our understanding in the biosynthetic mechanism of Er metabolites in YIM90600. EH, a novel erythronolide featuring an epoxy moiety, is supposed to be a shunt product from C-6/C-18 epoxidation and C-14 hydroxylation of 6-dEB. The *in vitro* enzymatic activity assay of EryF^Ac^ suggested the generation of a new metabolite with the molecular formula C_21_H_36_O_7_ (although the molecular ion current of which was significantly less than that of EB), supporting our assumption that EryF^Ac^, acting as a regiospecific P450 oxidase, is capable of catalyzing a successive two-step oxidation to form the epoxide of EH. However, subsequent *in vivo* genetic manipulation suggested that EryF^Ac^ could only catalyze one-step oxidation in *Sa. erythraea*. The *in vitro* enzymatic activity assay of EryK^Ac^ supported its role as a C-12 hydroxylase in generating Er-C and 3′-*N*-demethyl-Er-C, rather than a C-14 hydroxylase in EH formation. Although the biosynthesis of EH is still under discussion, exploration of the genetic background of YIM90600 and preliminary biosynthetic studies on its novel Er metabolites shall reveal the potential of extremophiles and shed new insights into Er biosynthetic pathways.

## Supporting Information

File S1 **This file contains Figure S1-Figure S14 and Table S1.** Figure S1. Construction and genotype verification of the *eryF^Sa^*-deleting mutant, *Sa. erythraea* EX101. (A) *Sa. erythraea* EX101 with a 771 bp deletion within *eryF^Sa^* is constructed via a double-crossover event. (B) Gel electrophoresis analysis of the PCR products amplified from the genomic DNAs of *Sa. erythraea* ZL2001 (lane 1), EX101 (lane 2), and the single-crossover exconjugant (lane 3), using primer pair pFf/pFr. Figure S2. Construction and genotype verification of the *eryBV^Sa^*-deleting mutant, *Sa. erythraea* EX102. (A) *Sa. erythraea* EX102 with a 786 bp deletion within *eryBV^Sa^* is constructed via a double-crossover event. (B) Gel electrophoresis analysis of the PCR products amplified from the genomic DNAs of *Sa. erythraea* ZL2001 (lane 1), EX102 (lane 2), and the single-crossover exconjugant (lane 3), using primer pair pBVf/pBVr. Figure S3. Construction of the gene complementation mutant, *Sa. erythraea* EX103. The gene fragment P_ermE_*-eryF^Ac^ is introduced into the artificial *attB* sites of *Sa. erythraea* EX101 via the actinophage ΦC31 integrase-mediated site-specific recombination. Figure S4. SDS-PAGE analysis of the purified recombinant proteins with 6 x His-tag at the *N* terminus. Recombinant EryF^Sa^ (lane 1) exhibited a molecular mass of 47.4 kDa, recombinant EryF^Ac^ (lane 2) exhibited a molecular mass of 47.6 kDa, recombinant EryK^Sa^ (lane 3) exhibited a molecular mass of 46.0 kDa, and recombinant EryK^Ac^ (lane 4) exhibited a molecular mass of 46.3 kDa. Figure S5. CO difference spectra of the cytochrome P450 oxidases, EryF^Sa^, EryF^Ac^, EryK^Sa^ and EryK^Ac^. UV-vis absorbance of both EryF^Sa^ (A) and EryF^Ac^ (B) exhibits a Soret peak at 423 nm under reducing condition, which shifts to 448 nm after binding of CO. UV-vis absorbance of both EryK^Sa^ (C) and EryK^Ac^ (D) exhibits a Soret peak at 420 nm under reducing condition, which shifts to 448 nm after binding of CO. Figure S6. HR-ESI-MS analyses of 6-dEB, EB, and the proposed 6, 18-epoxy-EB. A, purified 6-dEB with a molecular formula as C_21_H_38_O_6_, showing [M+Na]^+^ at m/z 409.2579, B, purified EB with a molecular formula as C_21_H_38_O_7_, showing [M+Na]^+^ at m/z 425.2517, C, an EryF^Ac^-catalyzed enzymatic reaction containing a compound with a molecular formula as C_21_H_36_O_7_, showing [M+Na]^+^ at m/z 423.2342. Figure S7. Proposed fragmentation scheme for 6-dEB and the ESI-MS-MS product ion spectrum of 6-dEB. Figure S8. Proposed fragmentation scheme for EB and the ESI-MS-MS product ion spectrum of EB. Figure S9. Substrate binding spectra for 6-dEB bound to the cytochrome P450 oxidases, EryF^Sa^ and EryF^Ac^. UV-vis absorbance of both EryF^Sa^ (A) and EryF^Ac^ (B) exhibits a Soret peak at 423 nm (green), which shifts to 392 nm after the addition of 6-dEB (blue). The Soret peaks at 392 nm increase with higher concentration of 6-dEB dissolved in the protein solutions (red). Figure S10. HPLC-ESI-MS analysis of the fermentation culture of *Sa. erythraea* ZL2001. Total ion current chromatogram (*i*), and reconstructed base peak chromatograms for Er-A (*ii*), Er-B (*iii*), Er-C (*iv*), and Er-D (*v*) are recorded. Figure S11. HPLC-ESI-MS analysis of the fermentation culture of *Sa. erythraea* EX101. Total ion current chromatogram (*i*), and reconstructed base peak chromatograms for 6-deoxy-Er-A (*ii*), 6-deoxy-Er-B (*iii*), 6-deoxy-Er-C (*iv*), and 6-deoxy-Er-D (*v*) are recorded. Figure S12. HPLC-ESI-MS analysis of the fermentation culture of *Sa. erythraea* EX102. Total ion current chromatogram (*i*), and reconstructed base peak chromatograms for 5-*O*-desosaminyl-EB (*ii*), 12-hydroxyl-5-*O*-desosaminyl-EB (*iii*) are recorded. Figure S13. HPLC-ESI-MS analysis of the fermentation culture of *Sa. erythraea* EX103. Total ion current chromatogram (*i*), and reconstructed base peak chromatograms for EH (*ii*), 6, 18-epoxy-EB (*iii*) are recorded. Figure S14. HPLC-ESI-MS analyses of the *in vitro* enzymatic reactions catalyzed by EryF^Ac^ and EryK^Ac^, and by EryF^Sa^ and EryK^Sa^, respectively. (A) Total ion current chromatogram (*i*) and reconstructed base peak chromatogram for EB (*ii*), 6, 18-epoxy-EB (*iii*), 12-hydroxyl-EB (*iv*), EH (*v*) of the EryF^Ac^ and EryK^Ac^ reaction mixture. (B) Total ion current chromatogram (*i*) and reconstructed base peak chromatogram for EB (*ii*), 6, 18-epoxy-EB (*iii*), 12-hydroxyl-EB (*iv*), EH (*v*) of the EryF^Sa^ and EryK^Sa^ reaction mixture. Table S1. Primers used for genetic manipulation and protein expression in this study.
(DOC)
