# Supplementary material for: Identification and Characterization of a New Erythromycin Biosynthetic Gene Cluster in Actinopolyspora erythraea YIM90600, a Novel Erythronolide-Producing Halophilic Actinomycete Isolated from Salt Field
Source: PLoS One. 2014 Sep 24;9(9):e108129. doi: 10.1371/journal.pone.0108129 (PMC4176971; doi:10.1371/journal.pone.0108129)
Supplement: File S1 — This file contains Figure S1-Figure S14 and Table S1. Figure S1. Construction and genotype verification of the eryFSa-deleting mutant, Sa. erythraea EX101. (A) Sa. erythraea EX101 with a 771 bp deletion within eryFSa is constructed via a double-crossover event. (B) Gel electrophoresis analysis of the PCR products amplified from the genomic DNAs of Sa. erythraea ZL2001 (lane 1), EX101 (lane 2), and the single-crossover exconjugant (lane 3), using primer pair pFf/pFr. Figure S2. Construction and genotype verification of the eryBVSa-deleting mutant, Sa. erythraea EX102. (A) Sa. erythraea EX102 with a 786 bp deletion within eryBVSa is constructed via a double-crossover event. (B) Gel electrophoresis analysis of the PCR products amplified from the genomic DNAs of Sa. erythraea ZL2001 (lane 1), EX102 (lane 2), and the single-crossover exconjugant (lane 3), using primer pair pBVf/pBVr. Figure S3. Construction of the gene complementation mutant, Sa. erythraea EX103. The gene fragment PermE*-eryFAc is introduced into the artificial attB sites of Sa. erythraea EX101 via the actinophage ΦC31 integrase-mediated site-specific recombination. Figure S4. SDS-PAGE analysis of the purified recombinant proteins with 6 x His-tag at the N terminus. Recombinant EryFSa (lane 1) exhibited a molecular mass of 47.4 kDa, recombinant EryFAc (lane 2) exhibited a molecular mass of 47.6 kDa, recombinant EryKSa (lane 3) exhibited a molecular mass of 46.0 kDa, and recombinant EryKAc (lane 4) exhibited a molecular mass of 46.3 kDa. Figure S5. CO difference spectra of the cytochrome P450 oxidases, EryFSa, EryFAc, EryKSa and EryKAc. UV-vis absorbance of both EryFSa (A) and EryFAc (B) exhibits a Soret peak at 423 nm under reducing condition, which shifts to 448 nm after binding of CO. UV-vis absorbance of both EryKSa (C) and EryKAc (D) exhibits a Soret peak at 420 nm under reducing condition, which shifts to 448 nm after binding of CO. Figure S6. HR-ESI-MS analyses of 6-dEB, EB, and the proposed 6, 18- [file pone.0108129.s001.doc]

**Identification and Characterization of a New Erythromycin Biosynthetic Gene Cluster in *Actinopolyspora* *erythraea* YIM90600, a Novel Erythronolide-producing Halophilic Actinomycete Isolated from Salt Field**

Dandan Chen^1,2^, Junyin Feng^2^, Lei Huang^1^, Qinglin Zhang^2^, Jiequn Wu^2^, Xiangcheng Zhu^3,4^, Yanwen Duan^3,4^, and Zhinan Xu^1^,*

Department of Chemical and Biological Engineering, Zhejiang University, 38 Zheda Road, Hangzhou 310027, China^1^; Huzhou Center of Bio-synthetic Innovation, Shanghai Institute of Organic Chemistry, Chinese Academy of Sciences, 1366 Hongfeng Road, Huzhou 313000, China^2^; Hunan Engineering Research Center of Combinatorial Biosynthesis and Natural Product Drug Discovery, Changsha, Hunan 410329, China^3^; Xiangya International Academy of Translational Medicine, Central South University, Changsha, Hunan 410013, China^4^.

* To whom correspondence should be addressed: Department of Chemical and Biological Engineering, Zhejiang University, 38 Zheda Road, Hangzhou 310027, China. Tel: 86-571-87951220, Fax: 86-571-87951220, Email: znxu@zju.edu.cn.

* To whom correspondence should be addressed: Department of Chemical and Biological Engineering, Zhejiang University, 38 Zheda Road, Hangzhou 310027, China. Tel: 86-571-87951220, Fax: 86-571-87951220, Email: znxu@zju.edu.cn.

Figure S1. Construction and genotype verification of the *eryF^Sa^*-deleting mutant, *Sa. erythraea* EX101. (A) *Sa. erythraea* EX101 with a 771 bp deletion within *eryF^Sa^* is constructed via a double-crossover event. (B) Gel electrophoresis analysis of the PCR products amplified from the genomic DNAs of *Sa. erythraea* ZL2001 (lane 1), EX101 (lane 2), and the single-crossover exconjugant (lane 3), using primer pair pFf/pFr.

Figure S2. Construction and genotype verification of the *eryBV^Sa^*-deleting mutant, *Sa. erythraea* EX102. (A) *Sa. erythraea* EX102 with a 786 bp deletion within *eryBV^Sa^* is constructed via a double-crossover event. (B) Gel electrophoresis analysis of the PCR products amplified from the genomic DNAs of *Sa. erythraea* ZL2001 (lane 1), EX102 (lane 2), and the single-crossover exconjugant (lane 3), using primer pair pBVf/pBVr.

Figure S3. Construction of the gene complementation mutant, *Sa. erythraea* EX103. The gene fragment *P_ermE_*-eryF^Ac^* is introduced into the artificial *attB* sites of *Sa. erythraea* EX101 via the actinophage ΦC31 integrase-mediated site-specific recombination.

Figure S4. SDS-PAGE analysis of the purified recombinant proteins with 6 x His-tag at the *N* terminus. Recombinant EryF^Sa^ (lane 1) exhibited a molecular mass of 47.4 kDa, recombinant EryF^Ac^ (lane 2) exhibited a molecular mass of 47.6 kDa, recombinant EryK^Sa^ (lane 3) exhibited a molecular mass of 46.0 kDa, and recombinant EryK^Ac^ (lane 4) exhibited a molecular mass of 46.3 kDa.

Figure S5. CO difference spectra of the cytochrome P450 oxidases, EryF^Sa^, EryF^Ac^, EryK^Sa^ and EryK^Ac^. UV-vis absorbance of both EryF^Sa^ (A) and EryF^Ac^ (B) exhibits a Soret peak at 423 nm under reducing condition, which shifts to 448 nm after binding of CO. UV-vis absorbance of both EryK^Sa^ (C) and EryK^Ac^ (D) exhibits a Soret peak at 420 nm under reducing condition, which shifts to 448 nm after binding of CO.

Figure S6. HR-ESI-MS analyses of 6-dEB, EB, and the proposed 6, 18-epoxy-EB. A, purified 6-dEB with a molecular formula as C_21_H_38_O_6_, showing [M+Na]^+^ at m/z 409.2579, B, purified EB with a molecular formula as C_21_H_38_O_7_, showing [M+Na]^+^ at m/z 425.2517, C, an EryF^Ac^-catalyzed enzymatic reaction containing a compound with a molecular formula as C_21_H_36_O_7_, showing [M+Na]^+^ at m/z 423.2342.

Figure S7. Proposed fragmentation scheme for 6-dEB [1] and the ESI-MS-MS product ion spectrum of 6-dEB.

Figure S8. Proposed fragmentation scheme for EB [1] and the ESI-MS-MS product ion spectrum of EB.

Figure S9. Substrate binding spectra for 6-dEB bound to the cytochrome P450 oxidases, EryF^Sa^ and EryF^Ac^. UV-vis absorbance of both EryF^Sa^ (A) and EryF^Ac^ (B) exhibits a Soret peak at 423 nm (green), which shifts to 392 nm after the addition of 6-dEB (blue). The Soret peaks at 392 nm increase with higher concentration of 6-dEB dissolved in the protein solutions (red).

Figure S10. HPLC-ESI-MS analysis of the fermentation culture of *Sa. erythraea* ZL2001. Total ion current chromatogram (*i*), and reconstructed base peak chromatograms for Er-A (*ii*), Er-B (*iii*), Er-C (*iv*), and Er-D (*v*) are recorded.

Figure S11. HPLC-ESI-MS analysis of the fermentation culture of *Sa. erythraea* EX101. Total ion current chromatogram (*i*), and reconstructed base peak chromatograms for 6-deoxy-Er-A (*ii*), 6-deoxy-Er-B (*iii*), 6-deoxy-Er-C (*iv*), and 6-deoxy-Er-D (*v*) are recorded.

Figure S12. HPLC-ESI-MS analysis of the fermentation culture of *Sa. erythraea* EX102. Total ion current chromatogram (*i*), and reconstructed base peak chromatograms for 5-*O*-desosaminyl-EB (*ii*), 12-hydroxyl-5-*O*-desosaminyl-EB (*iii*) are recorded.

Figure S13. HPLC-ESI-MS analysis of the fermentation culture of *Sa. erythraea* EX103. Total ion current chromatogram (*i*), and reconstructed base peak chromatograms for EH (*ii*), 6, 18-epoxy-EB (*iii*) are recorded.

Figure S14. HPLC-ESI-MS analyses of the *in vitro* enzymatic reactions catalyzed by EryF^Ac^ and EryK^Ac^, and by EryF^Sa^ and EryK^Sa^, respectively. (A) Total ion current chromatogram (*i*) and reconstructed base peak chromatogram for EB (*ii*), 6, 18-epoxy-EB (*iii*), 12-hydroxyl-EB (*iv*), EH (*v*) of the EryF^Ac^ and EryK^Ac^ reaction mixture. (B) Total ion current chromatogram (*i*) and reconstructed base peak chromatogram for EB (*ii*), 6, 18-epoxy-EB (*iii*), 12-hydroxyl-EB (*iv*), EH (*v*) of the EryF^Sa^ and EryK^Sa^ reaction mixture.

Table S1. Primers used for genetic manipulation and protein expression in this study.

| ***Name*** | ***Sequence*** | ***Description*** |
| --- | --- | --- |
| pFLf  pFLr | CCCCAAGCTTGCCGCGGTGCGGGCGGTGACCAG (*Hin*dIII)  GCTCTAGAGACCTCCACGCCCGGGTACTTCTTC (*Xba*I) | Cloning of the upstream region of *eryF^Sa^* for in-frame deletion |
| pFRf  pFRr | GCTCTAGAGACCCCCACCGCTTCGACGTCACCCGC (*Xba*I)  CGGAATTCGCTCGACTCCGCGCTGAAGGCGTCCG (*Eco*RI) | Cloning of the downstream region of *eryF^Sa^* for in-frame deletion |
| pBVLf  pBVLr | CCCCAAGCTTACAGCGCGGCGGCGCGCGCGGCGTC (*Hin*dIII)  GCTCTAGAGAAGGAGTAGCGGTGCACCTGAGCG (*Xba*I) | Cloning of the upstream region of *eryBV^Sa^* for in-frame deletion |
| pBVRf  pBVRr | GCTCTAGAGCACATGAATGGGATTGCATGCTACG (*Xba*I)  CGGAATTCAGCGGGCGGCCGCGCTGCACGAACTC (*Eco*RI) | Cloning of the downstream region of *eryBV^Sa^* for in-frame deletion |
| pFf  pFr | ATCTCGCCGGCGGTGCCCAGGG  TGGTGCTGCCCTGGATCTACCGC | Genotype verification of the *eryF^Sa^*-deleting mutant |
| pBVf  pBVr | ACCCGCCCTCGTACATCCCTGCTC  GGCCAACGACTTCCGCAGCGAC | Genotype verification of the *eryBV^Sa^*-deleting mutant |
| pAcF-Cf  pAcF-Cr | CCAAGCTTGCGGGCGGCGGAGAACTGATC (*Hin*dIII)  GCTCTAGACCGTAGCCAGGTGCTCATCCG (*Xba*I) | Cloning of the *eryF^Ac^* gene fragment with its terminator |
| pSaFf  pSaFr | TCATATGATGACGACCGTTCCCGATCTCGAAAG (*Nde*I)  TGAATTCTCATCCGTCGAGCCGCACCGGTAG (*Eco*RI) | Cloning of *eryF^Sa^* for protein expression in a heterologous host |
| pAcFf  pAcFr | TCATATGATGACGACCGTTCCCGACCTC (*Nde*I)  TGAATTCTCATCCCTCCAGTCGCACCGG (*Eco*RI) | Cloning of *eryF^Ac^* for protein expression in a heterologous host |
| pSaKf  pSaKr | TCATATGTTGACCACCATCGACGAAGTTC (*Nde*I)  TGAATTCCTACGCCGACTGCCTCGGCGA (*Eco*RI) | Cloning of *eryK^Sa^* for protein expression in a heterologous host |
| pAcKf  pAcKr | TCATATGTTGACCACTATCGACGAAGTCC (*Nde*I)  TGAATTCCTACGCCGACTGCGTCGACGA (*Eco*RI) | Cloning of *eryK^Ac^* for protein expression in a heterologous host |

Reference of Supporting Information

1. Roddis M, Gates P, Roddis Y, Staunton J (2002) Structural elucidation studies on 14- and 16-membered macrolide aglycones by accurate-mass electrospray sequential mass spectrometry. J Am Soc Mass Spectrom 13: 862-874.
